# Modeling the progression of Type 2 diabetes with underlying obesity

**DOI:** 10.1371/journal.pcbi.1010914

**Published:** 2023-02-27

**Authors:** Boya Yang, Jiaxu Li, Michael J. Haller, Desmond A. Schatz, Libin Rong

**Affiliations:** 1 Department of Mathematics, University of Florida, Gainesville, Florida, United States of America; 2 Department of Mathematics, University of Louisville, Louisville, Kentucky, United States of America; 3 Department of Pediatrics, University of Florida, Gainesville, Florida, United States of America; University of Michigan, UNITED STATES

## Abstract

Environmentally induced or epigenetic-related beta-cell dysfunction and insulin resistance play a critical role in the progression to diabetes. We developed a mathematical modeling framework capable of studying the progression to diabetes incorporating various diabetogenic factors. Considering the heightened risk of beta-cell defects induced by obesity, we focused on the obesity-diabetes model to further investigate the influence of obesity on beta-cell function and glucose regulation. The model characterizes individualized glucose and insulin dynamics over the span of a lifetime. We then fit the model to the longitudinal data of the Pima Indian population, which captures both the fluctuations and long-term trends of glucose levels. As predicted, controlling or eradicating the obesity-related factor can alleviate, postpone, or even reverse diabetes. Furthermore, our results reveal that distinct abnormalities of beta-cell function and levels of insulin resistance among individuals contribute to different risks of diabetes. This study may encourage precise interventions to prevent diabetes and facilitate individualized patient treatment.

## Author summary

Mathematical models depicting the progression of Type 2 diabetes can be used to study the durable effect of anti-diabetic agents at different disease stages. However, few existing models have been capable of depicting the long-term dynamics of diabetes progression. Mounting evidence has indicated that hyperinsulinemia aggravates insulin resistance no matter in which order they occur. We developed a generalized model which incorporates the effect of hyperinsulinemia on insulin resistance and enables the quantitative study of the impact of diverse diabetogenic factors on beta-cell dysfunction. Specifying the diabetogenic factor to be obesity-related in the modeling framework, we generated an obesity-related diabetes model to investigate the influence of obesity on the glucose regulatory system. The long-course glucose and insulin trajectories depicted by the obesity-diabetes model are consistent with clinical observations and capture the trend of the longitudinal glucose data of the Pima Indian tribe. This work may have the potential to help develop therapeutic strategies for diabetes.

## Introduction

Mathematical modeling has been a valuable approach to delineating the glucose metabolic system. Various mathematical models have been proposed over the last decades to study type 2 diabetes mellitus (T2D) focusing on different aspects [1–7]. A well-accepted model depicting the beta-cell mass dynamics along with the progression of T2D is developed by Topp et al. [1]. Topp’s model became a foundation for other models to study diabetes progression. To describe the effects of treatment on the time-course of insulin sensitivity and progressively impaired beta-cell function, De Winter et al. [2] developed a population pharmacodynamic model that consists of the differential equations of glucose, insulin, and glycosylated hemoglobin A1c. In [3], Gaetano et al. formulated a model of pancreatic islet compensation to depict the concurrent evolution of beta-cell mass, pancreatic beta-cell replication reserve, glycemia, and insulinemia. Wang studied the hypothesis that intermittent insulin secretion allows beta-cells to rest and be re-sensitized [4]. To explain the observation that fasting hyperinsulinemia can precede hyperglycemia by up to decades, Ha, Sherman, and their colleagues extended Topp’s model by relating beta-cell proliferation to the secretory workload of beta-cells [5], which can amplify the impact of small changes of glucose. The model successfully demonstrated the effect of weight loss and bariatric surgery on the glucose regulatory system. Despite all these efforts, a model that describes a long-course evolution of diabetes integrating diabetogenic factors (e.g. obesogenic environment) remains lacking.

**Fig 1:**
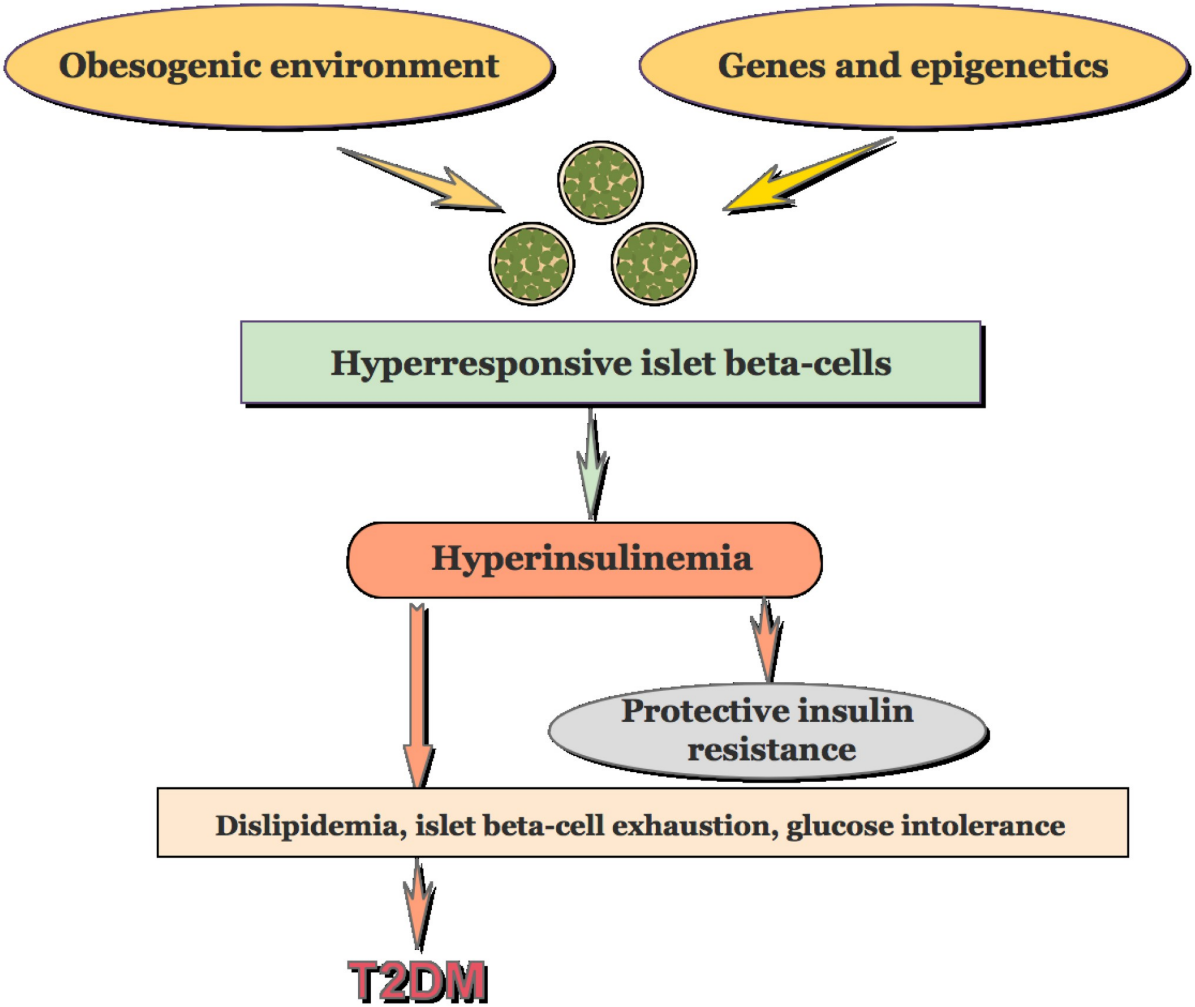
A conceptual framework that views insulin hypersecretion as the upstream of insulin resistance and the development of T2D. Different from the view that hyperinsulinemia is the downstream of insulin resistance, it proposes that hyperinsulinemia results from the hyperresponsive beta-cells to hostile environment, and insulin resistance is an adaptive mechanism protecting critical tissues from insulin-induced metabolic stress [13].

The primary causal factor for the progression of T2D has been believed to be insulin resistance induced by the interaction of insulin resistance genes and obesity which drives the hypersecretion of beta cells for insulin compensation. Esser et al. [8] remarked that this prevailing belief has since been modified: beta cell dysfunction, manifested as impaired insulin secretion, is the primary abnormality, with insulin resistance driving the hypersecretion of beta cells to maintain normal glucose levels. However, in the presence of an early beta cell dysfunction triggered by environmental factors and genetics, the increased beta cell secretory demand cannot be met by inadequate insulin, and dysglycemia occurs subsequently [8]. A recent and new perspective on T2D progression (Fig 1) claims that beta-cell hyper-responsiveness, induced by epigenetic-related or environmental factors (e.g. high-fat diets), causes hyperinsulinemia, with this aftereffect being the driving source of insulin resistance for individuals at risk of T2D [9–17]. The rationale behind this view is such that insulin resistance is a defense mechanism against insulin-induced metabolic stress rather than being harmful [13], as indicated by recent clinical studies in humans [18]. Johnson [18] and Corkey et al. [19] proposed hyperinsulinemia per se to be a manifestation of beta cell dysfunction including both the excessive and deficient insulin secretion stimulated by glucose as well as other nutrients. The mechanisms of T2D progression, including the order of each pathological stage, may vary among populations with different genotypes [18, 20]. *In vivo* studies have shown that hyperinsulinemia can cause insulin resistance for at least the phenotype of obesity-related diabetes [21–24]. Overall, surges of evidence indicate that hyperinsulinemia aggravates insulin resistance although the order of their occurrence remains under discussion and study [15, 25–27]. The field exploring the pathogenesis of T2D has come to the agreement on the critical role of beta-cell dysfunction in the progression to diabetes. Further understanding of the mechanisms underlying beta-cell dysfunction would contribute to better preventative and therapeutic interventions for the disease.

In this paper, we developed mathematical models to investigate the significance of beta-cell dysfunction on diabetes progression. We first considered which pathogenic factors lead to the dysfunction of beta-cells, as the glucose regulatory system of the human body can maintain homeostasis without external interference factors. The risks of beta-cell dysfunction and the development of T2D are related to sustained exposure to fuel excess, or the failure to store excess fuel properly [19], as well as the crosstalk interactions with other endocrine diseases [28]. Beta-cell function (total secretion of all beta-cells) and blood glucose levels are under the control of diverse hormones such as epinephrine, growth hormone, glucocorticoids, and thyroxine. Abnormal levels of these hormones can lead to an increased risk of hyperglycemia [29]. In consideration of these observations, we incorporated an interference factor into the glucose regulatory model, accounting for the progressive impact of the diabetogenic factor on the beta-cell function and the longitudinal regulation of glucose.

We formulated a base model for the undisturbed glucose-insulin regulatory system, upon which, we built the diabetes progression model framework with a generalized diabetogenic factor. We then specified the pathogenic factor to be obesity-related and construct the corresponding obesity-diabetes model. The simulated dynamics of the obesity-diabetes model delineate the long-term impact of obesity on the trends of glucose and insulin levels. We validated the model on the longitudinal data of the Pima Indian population, which characterizes the individualized glucose variations and predicts the glucose trajectories over their lifespan. Our model is capable of revealing distinct stages of the progression of diabetes (i.e., the euglycemic with hyperinsulinemia stage, prediabetic stage, and overt diabetic stage) and may assist physicians in designing optimal therapy with the individualized prediction of the disease evolution. Additionally, sensitivity tests on some key parameters of the obesity-related diabetes model were conducted to demonstrate the robustness of the model, and the analysis of the biological significance for these parameters sheds light on efficient treatment strategies for diabetes. We provide a holistic analysis of diabetes progression, including the identification of individualized obese thresholds of diabetogenic risk, the role of ethnic disparities in varied tolerance to the obesogenic environment, and the effectiveness of Roux-en-Y gastric bypass (RYGB) surgery. Thus, our modeling framework may be impactful to study individualized diabetic intervention and the effects of new therapies.

## Materials and methods

### Patient data

The patient data were obtained from the work by Mason et al. [30]. We studied 11 sets of data from Pima Indians of the Gila River Indian Community in Arizona, who took part in a longitudinal study of diabetes. The subjects over the age of 5 were invited every two years to examine their plasma glucose using a 75-g oral glucose tolerance test (OGTT). The selected subjects experienced no less than 10 non-diabetic biennial OGTT before developing hyperglycemia (2-h plasma glucose ≥ 200*mg*/*dl*). The OGTT measurements were taken before the initial diabetes diagnosis and the treatment. A majority of the time course glucose data are characterized by an initial linear trend accompanied with a sharp rise at the end (Figs 1, 3 in ref. [30]).

### A phenomenological diabetes progression model

The human body relies upon a compact control of blood glucose levels (70–100 mg/dl) to maintain its normal function. Insulin, synthesized in pancreas within the beta-cells, boosts the glucose utilization by target cells and plays an essential role in blood glucose regulation. High blood glucose levels induce the release of insulin from beta-cell secretory granules, which helps reduce the glucose concentration to the normal level. When the glucose level descends, the secretion of insulin ceases gradually. For an individual free from diabetogenic events, glucose homeostasis can be maintained by the glucose regulatory system. We first formulate a glucose-insulin regulation model for the euglycemic state, which serves as a base model for building our diabetes progression models. Very few models have accommodated the impact of hyperinsulinemia on insulin sensitivity [6]. We build the basic model upon the model of Topp et al. [1], where depicting the glucose-insulin dynamics in a slow time-scale (from days to years) becomes achievable by incorporating the functional pancreatic beta-cell mass equation into the system. The Topp model is

$$\begin{array}{cc} \frac{dG}{dt} & =G_{in}-g_{1}G-CGI,\\ \frac{dI}{dt} & =\frac{s_{1}G^{2}}{G^{2}+s_{2}}\beta -kI,\\ \frac{d\beta }{dt} & =(-d_{0}+r_{1}G-r_{2}G^{2})\beta , \end{array}$$

where *G* (mg/dl), *I* (*μ*U/ml), *β* (mg) stand for the plasma glucose concentration, insulin concentration and the mass of functional beta-cells (preserving appropriate insulin production and secretion) at time *t* (days), respectively. The parameter *G*_*in*_ stands for the average rate of glucose infusion per day (with meal ingestion as a main source), including the hepatic glucose production. The term *g*_1_*G* represents insulin-independent uptake of glucose, mainly by brain cells and nerve cells. In contrast, the term *CGI* depicts the insulin-dependent uptake of glucose, mostly by fat cells and muscle cells in the human body. In particular, the coefficient *C* (ml/*μ*U/day) stands for insulin sensitivity. The insulin secretion from beta-cells is assumed to be triggered by increased glucose levels in the form of the Hill function with coefficient 2, and the parameter *s*_1_ represents the secretory capacity per beta-cell. The insulin clearance rate is denoted by *k* (/day). The functional beta-cell mass is hypothesized to respond to glucose with a pattern similar to a downward parabola: moderate amount of glucose promotes the growth of beta-cells, while a high glucose level exacerbates beta-cell apoptosis, resulting in the decrease of functional beta-cell mass.

The Topp model is not sufficient to explain the occurrence of hyperinsulinemia before or during the prediabetic stage, since the unnoticeable change of glucose during these two stages cannot induce apparent increase of beta-cell mass [5]. Additionally, in Topp’s model [1], the insulin sensitivity *C* is assumed to be a constant for the entire life or an artificial time-dependent decreasing function *ae*^−*rt*^ with *a*, *r* > 0. Changing the value of insulin sensitivity *C* and the maximal releasing rate *s*_1_ in the model is still incapable of affecting the glucose level corresponding to the steady state [6]. This indicates that the Topp model cannot present the connection between varying degrees of impaired insulin signalling and the severity of hyperglycemia. To characterize the hyperinsulinemia-induced insulin resistance, we propose the insulin sensitivity to be a decreasing function of the insulin level, and assume that the insulin sensitivity function *C*(*I*) strictly decreases from *C*(0) to a positive number *r*_0_ when *I* increases. This is consistent with the finding of some studies that insulin sensitivity can be enhanced with reduced circulating insulin [31, 32].

Furthermore, in the Topp model, the glucose level is assumed to be an explicit factor regulating beta-cell mass. *In vivo* experiments have indicated that the replication of primary beta-cells is stimulated directly by insulin, instead of glucose, and the effects of glucose on beta-cell replication can be disclosed by glucose-induced insulin release and signaling [33, 34]. In particular, Johnson et al. proposed the “Sweet Spot” hypothesis, which speculates that moderately increased local insulin prompts the compensatory beta-cell hyperplasia, but once the elevated local insulin exceeds a certain level, it would cease to protect beta-cells and the beta-cell mass is anticipated to decrease [34]. Inspired by the formulation of the beta-cell equation in work [4], we model the net growth rate of functional beta-cell mass with a nonlinear function *f*_3_(*I*) that depends on the insulin level. In view of the net effect of beta-cell proliferation, neogenesis, and apoptosis on the growth rate of the functional beta-cell mass, *f*_3_(*I*) is assumed to take the form $f_{3}\left(I\right)=m_{1}I/\left(I^{2}+m_{2}^{2}\right)-m_{3}$, where *m*_1_, *m*_2_, *m*_3_ > 0. The function has two positive roots *I*_1_, *I*_2_ (*I*_1_ < *I*_2_) and *f*_3_(*I*) > 0 in the interval (*I*_1_, *I*_2_), while *f*_3_(*I*) < 0 when *I* > *I*_2_. With this function, when the insulin level exceeds the bound *I*_2_, the functional beta-cell mass will decrease to diminish the insulin secretion, dragging the insulin level down to *I*_2_ over time. That is, our base model is designed upon the assumption that the normal self-regulating function of beta-cells on insulin secretion maintains the homeostasis of insulin and glucose levels without interference factors from the hostile environment.

#### Model for undisturbed glucose-insulin regulatory system

The base model is given by

$$\begin{array}{c} \frac{dG}{dt}=G_{in}-f_{2}\left(G\right)-C\left(I\right)GI, \end{array}$$ (1)

$$\begin{array}{c} \frac{dI}{dt}=f_{1}\left(G\right)\beta -kI, \end{array}$$ (2)

$$\begin{array}{c} \frac{d\beta }{dt}=f_{3}\left(I\right)\beta , \end{array}$$ (3)

where *f*_2_(*G*) = *g*_1_*G*, $C\left(I\right)=r_{0}+\frac{r_{1}}{r_{2}+e^{r_{3}I}}$, $f_{1}\left(G\right)=\frac{s_{1}G^{2}}{G^{2}+s_{2}}$, $f_{3}\left(I\right)=\frac{m_{1}I}{I^{2}+{\left(m_{2}\right)}^{2}}-m_{3}$; all parameters in the model are positive.

Our rationale of incorporating the functional beta-cell mass into this model is to exhibit the long-course variation of beta-cell function, as we consider the integration of beta-cell secretory function (the ability to produce, store and release insulin) per cell and the functional beta-cell mass as an indicator of beta-cell function. When the normal regulation of the glucose-insulin system is disturbed, dysglycemia may occur, the progression of which can be elucidated by the diabetes progression models below.

#### A generalized diabetes progression model

The culprits of diabetes may vary for different subgroups of diabetic patients, which implies the distinction of possible interference factors to the glucose regulation system. Nevertheless, the underlying mechanisms through which the factors lead to dysglycemia are common. Numerous studies indicate that glycemia is primarily attributed to excess hepatic glucose output and abnormal insulin secretion and utilization [35]. Of note, beta-cell function is regulated by various mechanisms, not limited to glucose utilization [12]. Thus, confining the model for beta-cell function only with the variables of glucose and insulin may impede the study of beta-cell dysfunction. We aim to test through an *in-silico* approach how the T2D progression is affected by certain pathological factors. Here we propose a general form of diabetes progression model with a pathological factor *X* that is to be specified:

$$\begin{array}{c} \frac{dG}{dt}=G_{in}+p_{1}\left(X\right)-f_{2}\left(G\right)-C\left(I\right)GI, \end{array}$$ (4)

$$\begin{array}{c} \frac{dI}{dt}=f_{1}\left(G\right)p_{2}\left(X\right)\beta -kI, \end{array}$$ (5)

$$\begin{array}{c} \frac{d\beta }{dt}=(f_{3}\left(I\right)+p_{3}\left(X\right))\beta , \end{array}$$ (6)

where *X* is a bounded variable with a real value; all the variables in the system are in the time scale of days: *p*_1_(*X*) is incorporated into Eq (1) to stand for the increased hepatic glucose production caused by the pathological factor; *p*_2_(*X*) integrated into Eq (2) symbolizes the impact of the factor on the insulin secretion rate; *p*_3_(*X*) is incorporated to Eq (3) to describe the abnormal response of beta-cells to a hostile environment that develops in a slow time scale. The exact forms of the influence functions *p*_*i*_(*X*) (*i* = 1, 2, 3) will be determined with *X* being an obesity-related factor in Section. We assume that *p*_1_(*X*) = 0, *p*_2_(*X*) = 1, and *p*_3_(*X*) = 0 when *X* = 0 so the model is in accordance with the undisturbed glucose-insulin regulatory model when no diabetogenic factors exist in normal subjects.

### Obesity-related diabetes model

Environmental changes, including the changes of dietary habits and activity levels, are correlated to the epidemic incidence of diabetes [11, 36]. These changes interfere with the normal regulation of the glucose-insulin system [10, 12]. The upsurge in obesity has been closely linked to the increased prevalence of diabetes. Longitudinal studies have shown that the increase in the body mass index (BMI) over time is predominant among the risk factors for the raise in diabetes prevalence, while a mild decrease of BMI can induce a significant reduction in the risk of diabetes [37, 38]. Many mechanistic studies investigated the obesity-diabetes connection, but the view that obesity is the direct cause of diabetes is controversial [39]. Corkey et al. suggested that sustained exposure to excess fuel, or the failure of decreasing circulating fuel levels, stimulates and maintains basal hyperinsulinemia [19]. In addition, excessive calorigenic nutrients increase the production of reactive oxygen species (ROS) and the ectopic deposit of lipids in liver and pancreas, which may cause hepatic insulin resistance and beta-cell dysfunction [19, 40–43]. Although whether excess ROS and liver and pancreas fat are the primary causative factors for T2D has not been fully affirmed at the present stage, diabetes may share common pathogenic factors with obesity, at least for the subgroup of obesity-related diabetes [15]. We name the co-factor as the obesity-related factor hereinafter.

To quantitatively study the impact of the obesity-related factor on diabetes, we introduce the factor into the *GIβ* model. As the progression of obesity leads to a worse impact on the *GIβ* regulatory system, we set *X* to be the severity of the obese-related factor and quantify it as a variable with an upper bound of 1. Considering obesity is attributable to the progression of hepatic insulin resistance and gluconeogenesis [44], we design the elevated hepatic glucose production *p*_1_(*X*) to be a power function of *X*, which can be determined by the extent to which the pathogenic factor impacts the glucose generation rate. Prior research has established the association between obesity and a modest expansion of beta-cell mass in non-diabetic subjects [36]. In contrast, there is also a growing body of evidence that the accumulation of toxic metabolites (including ROS) within beta-cells in obese patients accelerates beta-cell apoptosis, leading to the progression to overt diabetes [45]. Thus, we assume functional beta-cell mass undergoes the impact of *X* in a pattern of a downward parabola and construct the influence function *p*_3_(*X*) with the form in Eq (9). Moreover, some experimental data demonstrate that insulin hypersecretion in obese subjects is attributed more to an increase in beta-cell secretory function than to an increase in beta-cell mass [36, 46]. For simplicity, we assume the beta-cell secretion function is linearly increasing with *X*, as shown in Eq (8). Furthermore, considering that obesity would progress slowly for an individual to a maximum limit, we assume *X* follows a logistic growth, given in Eq (10).

$$\begin{array}{c} p_{1}\left(X\right)=h_{1}X^{\alpha }, \end{array}$$ (7)

$$\begin{array}{c} p_{2}\left(X\right)=1+h_{2}X, \end{array}$$ (8)

$$\begin{array}{c} p_{3}\left(X\right)=q_{1}X\left(q_{2}-X\right), \end{array}$$ (9)

$$\begin{array}{c} \frac{dX}{dt}=n_{1}X\left(n_{2}-X\right). \end{array}$$ (10)

Here *h*_1_ represents the extent to which the gluconeogenesis rate is elevated by the factor; *α* determines how fast *p*_1_(*X*) changes with respect to *X*; *h*_2_*X* describes the increased insulin secretion rate induced by the factor; *p*_3_(*X*) stands for the influence of *X* on the net growth rate of functional beta-cell mass. The influence is positive when the values of *X* stay below *q*_2_, and the influence becomes negative when the level of *X* exceeds *q*_2_; that is, *q*_2_ describes the beta-cell tolerance for the pathogenic factor. The parameter *n*_1_ represents the growth rate of *X* in a specific environment; *n*_2_ stands for the maximal level of obesity that an individual would reach progressively (*n*_2_ ≤ 1). The *GIβ* dynamics may behave differently with varied parameter values. In particular, different parameter values in *C*(*I*) can reveal distinct insulin resistance profiles in the obesity-related diabetes group.

## Results

According to the American Diabetes Association (ADA), the fasting glucose levels for euglycemia, prediabetes, and diabetes are less than 100 mg/dl, 100 mg/dl to 125 mg/dl, and higher than 125 mg/dl, respectively [47]. The normal range of fasting insulin differs slightly between labs. Here we considered 5—20 *μ*U/mL as the reference range for normal fasting insulin and adopt *I* ≥ 25 *μ*U/mL as the criterion of hyperinsulinemia [48–50]. Throughout the numerical studies, we interpreted the simulation results according to the above ADA’s definition. The numerical method that solves the differential equations is the Runge-Kutta 4th-order algorithm embedded in the commercial software, Berkeley Madonna. The step size was set to be 0.005.

Using proper sets of parameter values for the undisturbed glucose-insulin regulatory model, as shown in Table 1, straightforward computation indicates the base model has a steady state (for fasting) at (*G*, *I*, *β*) = (100 mg/dl, 20 *μ*U/ml, 300). The parameter values and steady state may vary among individuals. In this work, excluding the values of *G*_*in*_, *g*_1_, *s*_2_, and *k*, we choose values of the other parameters to guarantee the consistency of the simulated glucose-insulin dynamics with clinical observations.

**Table 1:** Parameter values for the undisturbed glucose-insulin regulatory model and obesity-related diabetes model.

Parameters*	Set A	Set B	Unit	Source
*r*_0_	0.019	0.036	*ml* ⋅ *μU*^−1^ ⋅ *day*^−1^	see text
*r*_1_	1.98	29.32	*ml* ⋅ *μU*^−1^ ⋅ *day*^−1^	see text
*r*_2_	3.088	81.45	—	see text
*r*_3_	0.05	0.11	—	see text
*G*_*in*_	864	864	*mg* ⋅ *dl*^−1^ ⋅ *day*^−1^	[1]
*g*_1_	1.44	1.44	*day* ^−1^	[1]
*s*_1_	86.4	86.4	*μU* ⋅ *ml*^−1^ ⋅ *day*^−1^	see text
*s*_2_	20000	20000	*mg*^2^ ⋅ *dl*^−2^	[1]
*k*	432	432	*day* ^−1^	[1]
*m*_1_	0.1	0.1	*day* ^−1^	see text
*m*_2_	100	100	*μU* ⋅ *ml*^−1^	see text
*m*_3_	0.004	0.004	*day* ^−1^	see text
*h*_1_	300	300	*mg* ⋅ *dl*^−1^ ⋅ *day*^−1^	see text
*α*	$\frac{1}{3}$	$\frac{1}{3}$	—	see text
*h*_2_	0.1	0.1	—	see text
*q*_1_	0.04	0.025	*day* ^−1^	see text
*q*_2_	0.5	0.5	—	see text
*n*_1_	0.0005	0.0005	—	see text
*n*_2_	1	1	—	see text

The values of *r*_*i*_ (*i* = 0, 1, 2, 3) in set A manifest a higher insulin resistance level than the level signified by the values of *r*_*i*_ in set B; the larger value of *q*_1_ in set A represents a higher beta-cell susceptibility to the pathogenic factor compared with set B.

### Impact of the obesity-related factor on the progression of diabetes

**Fig 2:**
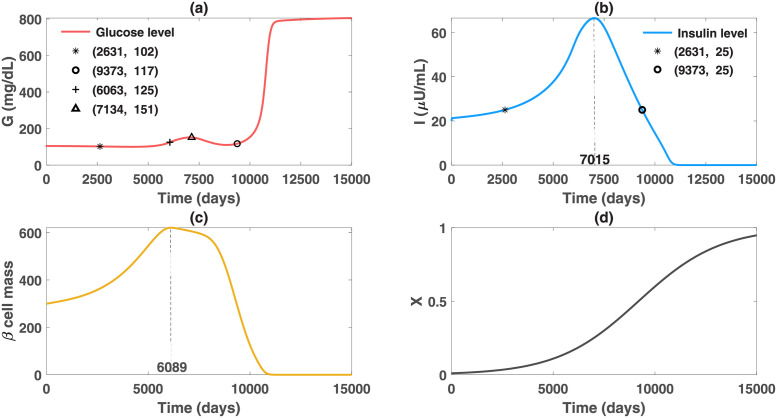
Dynamics of glucose, insulin and functional beta-cell mass level for patient A with severe obesity (*X* approaches 1 progressively). This figure depicts the impact of severe obesity on the glucose regulatory system. The pathogenic factor *X* initially overstimulates the beta-cell function of this patient. The insulin level exceeds 25 *μ*U/ml after day 2631, which represents the onset of hyperinsulinemia. However, at this time, the glucose level is 102 mg/dl, which is close to normal. As X continuously increases, the functional beta-cell mass keeps rising before day 6089 and then decreases afterwards. Correspondingly, the insulin level retains increasing before day 7015 and then descends. The hyperinsulinemic stage ends after day 9373 and insulin deficiency gradually occurs afterwards. In the hyperinsulinemic stage, patient A experiences some fluctuations in the glucose levels. The glucose level exceeds the threshold of diabetic stage on day 6063, yet decreasing for a while after reaching the level 151 mg/dl on day 7134. Subsequent to the appearance of beta-cell failure and insulin deficiency, a sharp rise of the glucose level occurs, transitioning patient A to overt diabetes.

We created a virtual patient A with the parameter values in set A of Table 1 and the initial condition of (*G*, *I*, *β*, *X*) = (100 mg/dl, 20 *μ*U/ml, 300, 0.01) at a fasting state. We further assume the obesity severity can gradually reach the upper bound of 1 over the lifespan of the virtual patient. The simulation results of the obesity-related diabetes model, shown in Fig 2, demonstrate that the obesity-related factor can lead to the elevation of the person’s insulin level and patient A would develop hyperinsulinemia in about 7 years (2631 days). During the first seven years, the glucose levels are below 102 mg/dl with slightly increased insulin levels. However, the insulin level rises higher and quicker in the first hyperinsulinemic stage, and the glucose level progressively increases to 125 mg/dl in 17 years (6063 days). The result that hyperinsulinemia precedes the onset of diabetes of patient A for 9.6 years, is consistent with the clinical phenomenon that fasting hyperinsulinemia, as a prediction of diabetes, may precede hyperglycemia by up to decades [51].

As the beta-cell function starts to fail but has not completely failed, due to the mitigated hyperinsulinemia, the insulin sensitivity gradually increases, which pauses the progression of the ultimate hyperglycemia and improves the glucose level from 151 mg/dl to 117 mg/dl. The fluctuations of glucose levels have also been observed in some clinical data (such as the Pima Indian data that will be presented later for data fitting in this paper). Nevertheless, insulin deficiency occurs over time subsequent to the occurrence of beta-cell failure. The massive and fast decline of insulin levels leads to the upsurge of glucose and transitions the patient to overt diabetes in the end (Fig 2).

**Fig 3:**
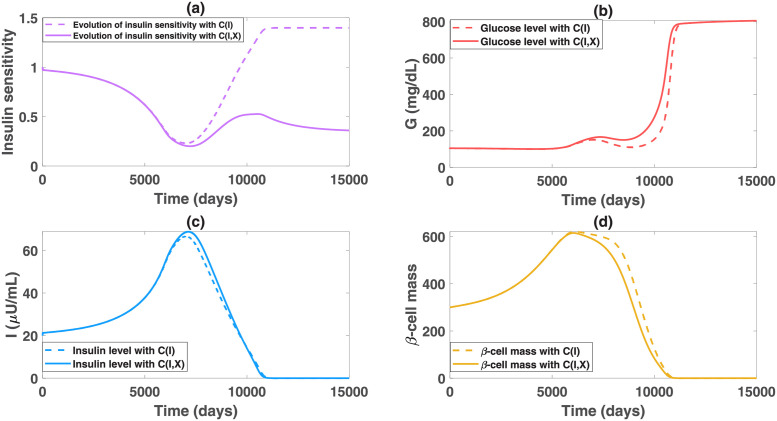
Comparison of the insulin sensitivity and *GIβ* dynamics using *C*(*I*) and *C*(*I*, *X*) in the model. The function *C*(*I*, *X*) is chosen to be $C\left(I\right)\cdot \left(1-\frac{0.8X^{4}}{X^{4}+0.5^{4}}\right)$, where *C*(*I*) is given in model (1). The other parameters maintain the same as in Fig 2. Graph (a) shows the time evolution of the insulin sensitivity *C*(*I*) in the dashed curve and *C*(*I*, *X*) in the solid curve. Graphs (b), (c), and (d) exhibit the corresponding *GIβ* dynamics under the two insulin sensitivity functions.

In the simulation results of Fig 2, we assume that the insulin sensitivity function *C*(*I*) depends only on the insulin level. The predicted dynamics of the insulin sensitivity are shown in Fig 3A (dashed line). Although a decreasing function *C*(*I*) can describe an enhanced insulin sensitivity with reduced circulating insulin, there is a limitation of using this function. As insulin decreases to a value lower than the initial level, insulin sensitivity is predicted to become greater than its initial value (Fig 3A dashed line). This seems to contradict the known physiology that insulin sensitivity decreases progressively with advancing diabetes and with obesity. To overcome this limitation, we assume that the insulin sensitivity function also depends on the obesity-related factor *X* (i.e. assuming *C*(*I*, *X*) decreases as *X* increases), accounting for the elevated insulin resistance driven by increased adiposity and free fatty acids [15, 52]. Choosing a specific function of *X*-induced insulin resistance, we plot the time evolution of insulin sensitivity *C*(*I*, *X*) in Fig 3A (solid line). Model simulation with the new function *C*(*I*, *X*) shows that the insulin sensitivity decreases before the onset of diabetes, then increases slightly for a while due to diminished insulin-induced resistance, and then goes back to the decreasing trend with the progression of obesity. This is consistent with the long-term behavior of insulin sensitivity. We also compare the dynamics of glucose, insulin and *β*-cell mass using the two functions *C*(*I*) and *C*(*I*, *X*) and find no significant difference in the *GIβ* dynamics (Fig 3B–3D, dashed vs. solid line). This result is not surprising as reduced insulin sensitivity only plays a significant role in transiting subjects from euglycemia to the onset of diabetes. In the diabetic stage, the defect of beta-cell function and mass is the dominant factor driving the deterioration of the disease [8]. For these reasons, we use the original function *C*(*I*) that minimizes the number of model parameters in later simulations and data fitting to the Pima Indian patients.

### Controlling/eradicating the obesity-related factor alleviates/reverses diabetes

**Fig 4:**
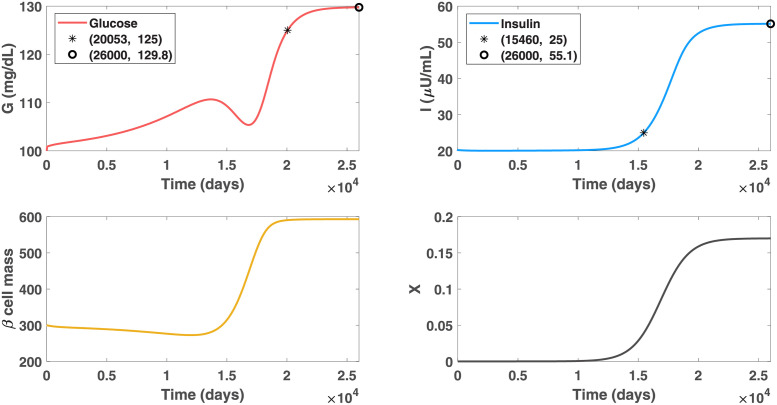
Dynamics of glucose, insulin and functional beta-cell mass level for patient A with mild obesity (*X* approaches 0.17 progressively). This figure describes the impact of the controlled obesity-related factor on the alleviation of diabetes. In contrast to the occurrence of beta-cell failure and subsequent insulin deficiency in Fig 2, hyperinsulinemia would occur to patient A on day 15460 and stay in the remainder of the life of this patient with undamaged pancreatic function. The glucose levels can be controlled below 125 mg/dl before day 20053, indicating a deferral of 38 years to develop diabetes compared with the condition in Fig 2. In addition, a moderate glycemic level, less than 130 mg/dl, can be maintained in the later life of this patient.

Distinct risk levels under the obesogenic environment, quantified by the different upper bound values of *X*, have varied influences on the *GIβ* system. Suppose that patient A is exposed to a mild obesogenic environment, where the maximal value that the obesity-related factor *X* would increase to is 0.17. The initial condition of *X* is set to be close to zero. The other parameters remain the same as those in Fig 2. The simulation results, presented in Fig 4, show that hyperinsulinemia would occur in 42 years (15460 days) and stay for the remainder of the life of patient A. Additionally, the patient would not become diabetic until 55 years (20053 days) later, a delay of 38 years relative to the condition of the uncontrolled obese severity in Fig 2. In contrast with the advanced diabetes that patient A would develop under the severe obesogenic environment, a moderate glycemic level of less than 130 mg/dl can remain in the later life of patient A with the alleviated obesity-related factor. The apparent alleviation of the disease progression rate and severity with the reduced level of obesity supports the recommendation of physicians for maintaining a healthy lifestyle.

We further investigated the evolution of diabetes with altered upper bound values of *X*, representing the varied maximal severities that the obesity-related factor can develop to. The results reveal 0.11 as the threshold value of the upper bound, separating the euglycemic and diabetic states for patient A. Below this threshold value, the *X*-induced beta-cell dysfunction is mild and the influence of moderately elevated insulin can be counteracted by temperate insulin resistance or glucagon to avoid the occurrence of hypoglycemia. On the other hand, when *X* exceeds 0.11 gradually, the excessive beta-cell secretory response leads to considerably high insulin, followed by severe insulin resistance and extra hepatic glucose production. There are two cases subsequent to this scenario. If the elevation of the *X* value stops at a moderate level, the expansion of beta cell mass would halt and the glucose can stay at a medium-high level. In the case that the *X* value continues to increase, the beta-cells cannot endure the excessive adverse pressure from the hostile environment, and initiate self-destruction. Consequently, beta-cell failure and insulin deficiency occur. The glucose would rise to an extremely high level, which can cause coma in certain extreme occasion.

**Fig 5:**
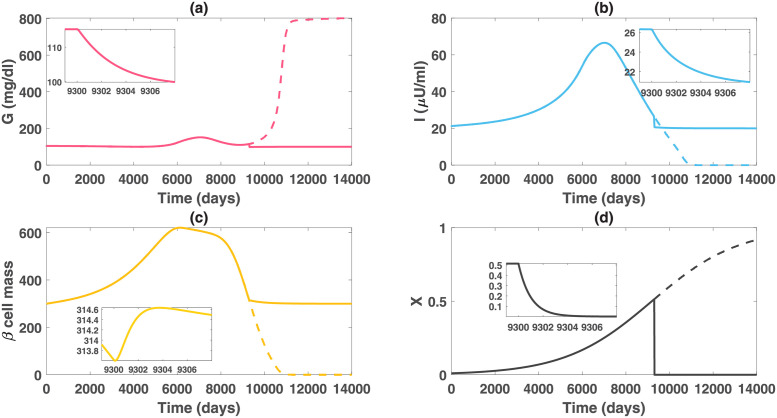
Altered dynamics of glucose, insulin and functional beta-cell mass level for patient A after taking the Roux-en-Y gastric bypass surgery. The patient is assumed to take the surgery on day 9300 and the obesity-related factor *X* is postulated to decrease exponentially to zero in approximately a week. The *GIβ* dynamics with taking the Roux-en-Y surgery are plotted in solid curves and the dashed curves represent the expected *GIβ* trajectories without taking the intervention. The solid curves are zoomed in to show the variations of the *GIβ* levels during the week after surgery. All of the other parameters here remain the same as those in Fig 2, except for those in the equation of *X*. Graph (a) illustrates that in contrast to the upsurge of glucose level after day 9300 without any intervention, the glucose levels are reduced to 100 mg/dl following surgery for one week. Graph (b) and (c) demonstrate the bariatric surgery can promptly halt the occurrence of beta-cell failure and insulin deficiency. Normal levels of insulin can be maintained after the first week.

Moreover, the obesity-related diabetes model can reveal the success of RYGB surgery in promptly reversing diabetes. Pories et al. proposed that the gastrointestinal diabetogenic signal of a patient disappears right after RYGB, followed by the correction of the fasting hyperinsulinemia [10]. We hypothesize that the obesity-related factor can be eradicated quickly from diabetic patients after the surgery. Suppose patient A takes the gastric bypass surgery on the 9300th day, that is, the intervention is performed before the patient would go through the severe diabetic stage. The factor is assumed to decrease approximately to zero in one week, as shown in Fig 5D. The simulation results illustrate that in contrast to the upsurge of glucose level after day 9300 without any intervention, the glucose levels are reduced to 100 mg/dl following surgery for one week. Meanwhile, the high insulin levels decline quickly and remain normal after the first week. These results are in agreement with the clinical data that show the correction of hyperinsulinemia and a significant reduction of fasting glucose even in the first week after surgery [53]. The study [53] suggests that the reduced glucose production, rather than the increased glucose disposal, contributes to the amelioration of diabetes after surgery. This effect is demonstrated in our model, as the extra hepatic glucose production *p*_1_(*X*) would be instantly removed along with the vanishment of the pathological factor. Moreover, the terminated decline of functional beta-cell mass after surgery, shown in Fig 5C, represents the improved beta-cell function. This underlies the improved postprandial insulin secretion for patients undergoing the surgery.

### Distinct levels of insulin resistance and beta-cell function contribute to different risks of diabetes

Ethnic distinction in the incidence of T2D has been recorded in the clinical literature. In particular, higher insulin resistance and up-regulated beta-cell function in African Americans, compared with non-Hispanic whites, are documented in most available data and are suggested to elevate the risk of T2D [54]. The underlying mechanism causing this disparity in the risks of diabetes may be explained by the obesity-related diabetes model.

**Fig 6:**
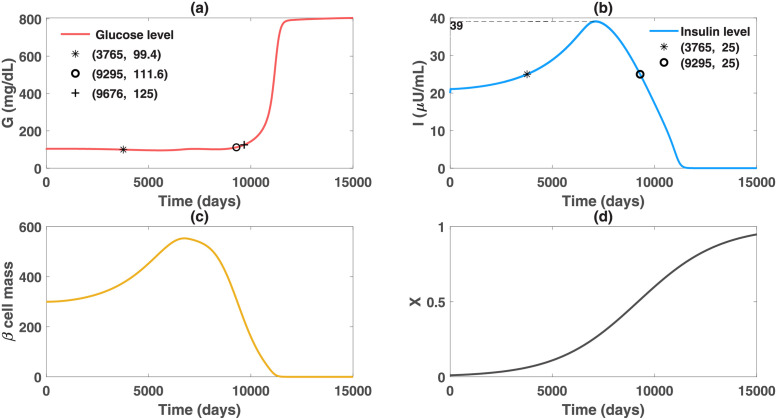
Dynamics of glucose, insulin and functional beta-cell mass for patient B with severe obesity (*X* approaches 1 progressively). Patient B is assumed to have a lower insulin resistance level and less beta-cell hyperresponsiveness to the obesity-related factor than patient A does. Graph (b) shows the hyperinsulinemic stage of patient B lasts for 15.2 years, which is 3.3 years shorter than patient A does in Fig 2. The maximal insulin level he would develop is 39 *μ*U/ml, which is 27.6 *μ*U/ml lower than the highest level that patient A attains. During this hyperinsulinemic stage, the glucose levels can be well controlled beneath 111.6 mg/dl. Additionally, patient B would not step into the diabetic stage until day 9676, which is 11.5 years later than patient A does.

We then selected a virtual patient B who has a lower insulin resistance level than patient A, and assume the obesity-related factor has a less up-regulating effect on the beta-cell function of patient B. The parameter setting for this patient is listed in the Set B of Table 1. We investigated the *GIβ* dynamics with an initial euglycemic state under the progressive severe obesogenic environment. Fig 6 shows that the hyperinsulinemic stage of patient B lasts 3.3 years (1212 days), shorter than patient A with the same obese severity, and the maximal insulin level he would reach is 27.6 *μ*U/mL, lower than the highest level that patient A attains. During this hyperinsulinemic stage, the glucose levels of patient B are well controlled beneath 111.6 mg/dl. Additionally, he would not step into the diabetic stage until 26.5 years (9676 days) afterwards, which is 11.5 years later than patient A does. The comparisons of diabetic progression between patient A and patient B theoretically exhibit the clinical observation that the risk and progression rate of developing diabetes varies with ethnic disparities, even if the cohorts are exposed under the same obesogenic environment.

**Fig 7:**
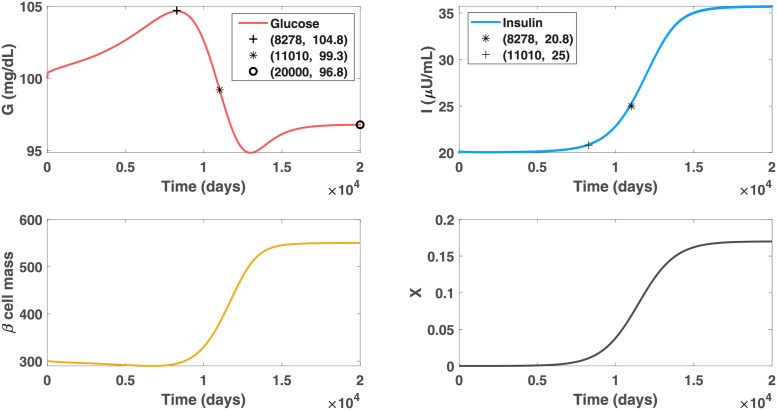
Dynamics of glucose, insulin and functional beta-cell mass level for patient B with mild obesity (*X* approaches 0.17 progressively). In this case, the factor *X* can only increase the glucose level to 104.8 mg/dl and the elevation is counteracted afterward by an upsurge of insulin. The insulin level remains moderately high after day 11010, driven by the continual hyperresponsiveness of beta-cells to the pathogenic risk. At the end, the glucose concentration of patient B can stay stable at a euglycemic level with 96.8 mg/dl.

We subsequently considered the *GIβ* dynamics of patient B in the mild obesogenic environment, where *X* gradually increases to the maximum value of 0.17 with an initial value close to zero. All the other parameter values remain the same as those in Fig 6. The simulation results, presented in Fig 7, illustrate that the mild obese-related factor would slightly increase the glucose level of patient B to 104.8 mg/dl but the elevation would be counteracted afterward by an upsurge of insulin. Although the glucose level would return to the normal reference range before the insulin level exceeds the hyperinsulinemic threshold 25 *μ*U/ml, the insulin level of patient B undergoes another elevation of 10.6 unit driven by the continual hyperresponsiveness of beta-cells to the increasing pathogenic risk. In the end, the glucose concentration would stay stable at a euglycemic level of 96.8 mg/dl. The different glucose dynamics compared with those in Figs 2, 4 and 6 suggest that the effects of a lower insulin resistance level, reduced up-regulated beta-cell function and mitigated obesogenic environment together contribute to the successful control of blood glucose. Moreover, the comparison of the *GIβ* dynamics between Figs 4 and 7 reveals that the difference in insulin resistance levels and beta-cell function among individuals can provide an explanation of the phenomenon that some individuals with mild obesity are able to remain euglycemic while others may not. Further numerical investigation exhibits the threshold value of *X* for patient B to develop diabetes is 0.41. The increase of the threshold value compared with the value of patient A, indicates that the lower insulin resistance and less up-regulated beta-cell response can increase the tolerance of patient B to the obesogenic environment.

### Sensitivity tests on parameters

We performed sensitivity tests on the key parameters *h*_1_, *α*, *h*_2_, *q*_1_, and *q*_2_ in the obesity-related diabetes model, and investigate the biological influence of these parameters on the disease progression. In the graphs (a)-(f) of Supplemental S1 Fig, we examined the effects of different parameter values in *p*_1_(*X*) on the *GIβ* dynamics. The results depict that the dynamics of insulin stay the same and the dynamics of glucose are slighted changed when the value of *h*_1_ is varied up to 10 percent. As the value of *α* changes up to 10 percent, the alterations of the *GIβ* dynamics are unnoticeable. Graphs (a), (b), and (c) in Supplemental S2 Fig demonstrate that the change of the *GIβ* dynamics is negligible when we vary *h*_2_, the *X*-induced insulin secretion rate, up to 10 percent. Graphs (a)-(f) in Supplemental S3 Fig depict the different *GIβ* dynamics where the parameter values in *p*_3_(*X*) are varied up to 10 percent. The shifts of the *GIβ* curves, corresponding to the different parameter values of *q*_1_ and *q*_2_ in graph (a)-(f), only represent the varied onset time and severity of diabetes. Overall, the variation of these parameter values has no impact on the analysis and general conclusions obtained from our modeling work.

Moreover, the sensitivity results reveal the impact of these parameters on the *GIβ* dynamics. Graphs (b) and (e) in Supplemental S3 Fig illustrate that the increase of *q*_1_, representing higher beta-cell susceptibility to the hostile environment, can reinforce hyperinsulinemia. Compared with the elevation of *q*_1_, the increase of *q*_2_, which stands for the enlarged tolerance of beta-cells to the pathological factor, contributes more to the high insulin level. The graphs (c) and (f) indicate that the influence of the factor *X* on beta-cells is not the sole determinant of functional beta-cell mass. When the increment of *q*_1_ or *q*_2_ exceeds a certain level, the insulin concentration would be significantly excessive, which instead prevents the expansion of functional beta-cell mass to slow down the insulin secretion. This reveals the regulation of functional beta-cell mass is one of the defensive approaches to reducing insulin-induced metabolic stress. Additionally, the graphs (f) and (d) demonstrate the increased beta-cell tolerance to the pathogenic factor would defer the occurrence of beta-cell failure and delay the onset of diabetes. We further make significant changes of the value of *q*_1_ to highlight its effect on the *GIβ* dynamics. The graph (g) demonstrates the considerable decrease of *q*_1_ can alleviate or even prevent the progression of hyperglycemia, which is enabled by the avoidance of beta-cell failure and insulin deficiency, as shown in the graphs (h) and (i). Further investigations on the significance of *h*_1_ are shown in the graphs (g) and (i) of Supplemental S1 Fig. These graphs illustrate that a substantial increase of *h*_1_ can speed up diabetes progression and elevate the steady state of the glucose level. This suggests that approaches to reducing hepatic gluconeogenesis are important in the treatment of hyperglycemia. Nevertheless, decreasing the value of *h*_1_ alone cannot prevent the development of hyperglycemia. In addition, although the variations of *h*_1_ have insignificant effects on the insulin dynamics, as shown in the graph (h), the dynamics of functional beta-cell mass alter with different values of *h*_1_. This reveals the indirect influence of glucose level on the regulation of functional beta-cell mass.

Graphs (d)-(f) in Supplemental S2 Fig exhibit the influence of significantly elevated *h*_2_ on the *GIβ* dynamics. Although the considerable increase of *h*_2_ contributes to an apparent up-shift of the insulin curve, the curve of the functional beta-cell mass shifts down in the meantime. That is, the factor-induced expansion of functional beta-cell mass would be diminished apparently when the factor-induced insulin secretion rate is elevated substantially in the same pathogenic environment. This suggests the mass and the insulin releasing function together represent the total function of beta-cells, and the beta-cells may reduce its reproductive effort when the insulin releasing function is enhanced.

Lastly, the severity of the pathogenic factor has substantial impact on the diabetes progression. In the obesity-related diabetes model, the dynamics of factor *X* are determined by the parameters *n*_1_ and *n*_2_. The comparison of the *GIβ* dynamics between Figs 4 and 7 has demonstrated the influence of the diminished value of *n*_2_ on disease alleviation or prevention. We further investigated the effect of *n*_1_ on the progression of hyperglycemia, as shown in Supplemental S4 Fig. These graphs exhibit that the significantly decreased value of *n*_1_, leads to a remarkably lowered value of *X* at each time point, inducing considerable alleviation or delay of the disease. Thus, mitigating the progression rate of the diabetogenic factor contributes to the amelioration of the disease.

### Best fits of the obesity-related diabetes model to Pima Indian data and its implication

We fitted *G*(*t*) in the obesity-related diabetes model to 11 sets of glucose data of the Pima Indians [30]. The data fitting was performed by the commercial software package Berkeley Madonna. An optimal set of parameters were determined from the best fitting, searching for the minimum root mean square (RMS) between the model prediction and the data with the following formula

$$RMS=\sqrt{\frac{\sum_{i=1}^{n}{\left[G\left(t_{i}\right)-\hat{G}\left(t_{i}\right)\right]}^{2}}{n}}$$

**Fig 8:**
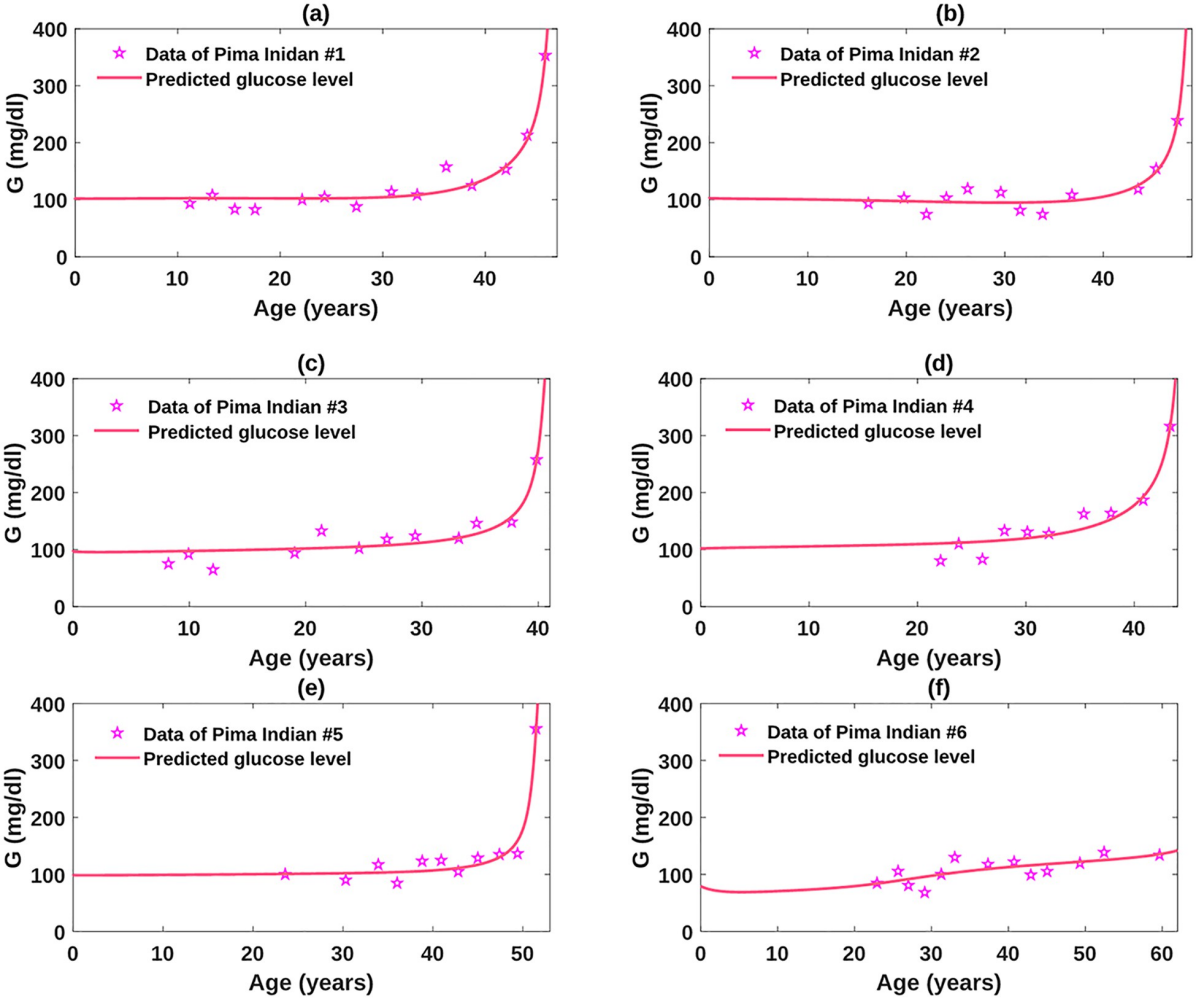
Obesity-related diabetes model fit to plasma glucose data for Pima Indian #1–#6. Overall, the glucose levels of these patients were steadily trending upwards.

**Fig 9:**
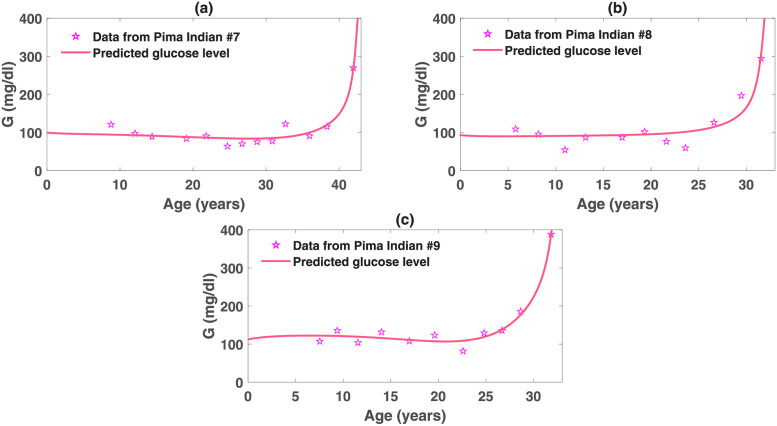
Obesity-related diabetes model fit to plasma glucose data for Pima Indian #7–#9. These data exhibit waveringly trends before the outbreak.

**Fig 10:**
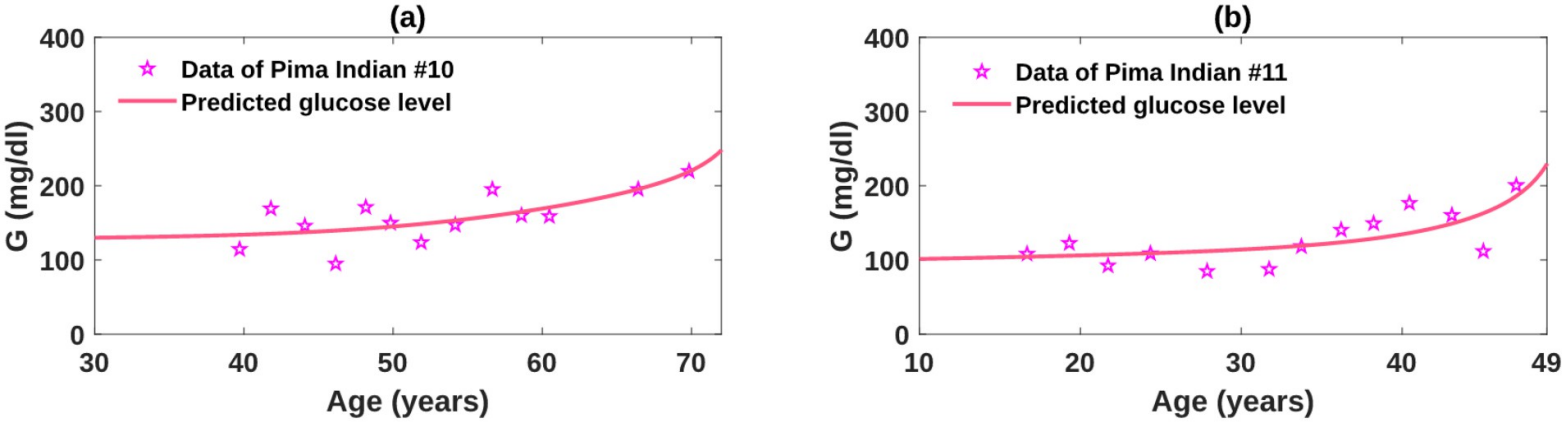
Obesity-related diabetes model fit to plasma glucose data with large fluctuations for Pima Indian #10 and #11.

In the formula, $\hat{G}\left(t_{i}\right)$ represents the glucose level at time *t*_*i*_ predicted by the model, and *G*(*t*_*i*_) is the corresponding data at *t*_*i*_. To avoid over-fitting, we kept all the parameter values used for the simulation of the base model except those in the insulin sensitivity function *C*(*I*), as the change of insulin sensitivity level has major influence on the disease progression. In addition, we fixed *n*_2_ to be 1 and relied on the variation of *n*_1_ to present different dynamics of *X* that patients may develop. The best fits, shown in Figs 8, 9 and 10, characterize the trend of glucose variations presented in the patients. In particular, each outbreak period displayed in the data sets is captured by our fits. The corresponding parameter values to the best fits are listed in Table 2. The estimated parameter values exhibit significant differences in distinct characteristics of the longitudinal T2D data, which are consistent with the parameter analysis results. Detailed explanations and possible biological mechanisms for these parametric variations are discussed below.

**Table 2:** Parameter values of the best fits of the obesity-related diabetes model to the glucose data of Pima Indian #1-#11.

Parameters	*r*_0_ (*ml* ⋅ *μU*^−1^ ⋅ *day*^−1^)	*r*_1_ (*ml* ⋅ *μU*^−1^ ⋅ *day*^−1^)	*r*_2_	*r*_3_	*h*_1_ (*mg* ⋅ *dl*^−1^ ⋅ *day*^−1^)	*q*_1_ (*day*^−1^)	*q*_2_	*n*_1_
Pima Indian #1 (Fig. 1A in ref. [30])	8.21 E-2	4.9001	3.0362	0.0286	500	0.0400	0.22369	2.5 E-4
Pima Indian #2 (Fig. 3A in ref. [30])	3.64 E-2	3.6947	1.7159	0.0367	202.13	0.0486	0.2803	2.47 E-4
Pima Indian #3 (Fig. 1B in ref. [30])	3.05 E-2	1.4051	1.0001	0.0466	383.79	0.0217	0.1000	2.86 E-4
Pima Indian #4 (Fig. 3E in ref. [30])	3.6 E-3	1.5116	2.4227	0.0178	500	0.0323	0.1001	2.44 E-4
Pima Indian #5 (Fig. 3B in ref. [30])	4.7 E-3	1.7210	1.2598	0.06	125.29	0.0166	0.100	2.32 E-4
Pima Indian #6 (Fig. 3F in ref. [30])	2.21 E-2	1.927	1.0499	0.03843	281.41	0.00126	0.15833	6.33 E-4
Pima Indian #7 (Fig. 1C in ref. [30])	1.93 E-2	1.4614	2.2877	0.03	100	0.0393	0.3007	2.94 E-4
Pima Indian #8 (Fig. 1D in ref. [30])	1.14 E-2	1.5516	1.2893	0.0424	217.01	0.0113	0.2161	4.47 E-4
Pima Indian #9 (Fig. 3C in ref. [30])	9.3 E-3	0.8520	1.5671	0.01	500	0.0193	0.5039	4.77 E-4
Pima Indian #10 (Fig. 1E in ref. [30])	3.6 E-3	1.3406	2.5324	0.0382	179.12	0.0027	0.3490	2.59 E-4
Pima Indian #11 (Fig. 1F in ref. [30])	1.8 E-2	1.2962	1.5275	0.0257	500	0.0087	0.1	2.58 E-4

Among the patient data sets, the data of Pima Indian #6 describe a euglycemic level, and the data of patient #10 and 11 depict relatively well-controlled glucose levels. Which parameters play a key role in the disease control and prevention? A thorough comparison of the parameter values among the Pima Indian patients reveals that the small value of parameter *q*_1_, representing reduced susceptibility of beta-cells to the hostile environment, is vital for the control of diabetes. We further compared the parametric differences within the Pima Indian #6, 10, and 11. The Pima Indian #6 had higher insulin sensitivity and lower *q*_1_ than Pima Indian #10 and 11. However, the estimated value of *n*_1_ for Pima Indian #6 is considerably larger than those for Pima Indian #10 and 11, which implies Pima Indian #6 was exposed under a worse diabetogenic environment. This indicates a strong defensive function of beta-cells can counteract the adverse impact of diabetogenic factors, contributing to the disease prevention. Thus, treatment approaches to suppress the detrimental effect of the diabetogenic factors on the beta-cells may be more efficient than the efforts to improve the hostile environment, such as exercise and diet control. In addition, compared with Pima Indian #10, Pima Indian #11 who had higher insulin sensitivity, exhibited higher rate of glucose elevation with larger values of *q*_1_, *q*_2_, *h*_1_. This suggests the monotherapy targeting insulin resistance may not be sufficient to cure diabetes.

We further investigated the possible factors driving Pima Indian #8 and #9 to develop diabetes significantly earlier than other Pima Indian patients. The clear distinction between the parameter values of Pima Indian #8, #9 and other patients only lies in the parameter *n*_1_. That is, Pima Indian #8 and #9 had higher values of *n*_1_ than other patients, except for the value of Pima Indian #6. The revealed influence of *n*_1_ on diabetic progression is in line with the result of parameter analysis for *n*_1_ (Supplemental S4 Fig), which provides theoretical evidence for employing the approaches to ameliorate the hostile environment as a treatment strategy. In addition, the impact of the high *n*_1_ value on the glucose regulatory system of Pima Indian #6 was offset by the remarkably low *q*_1_ value. This observation is consistent with the clinical phenomenon that some individuals are able to maintain euglycemic although they are exposed to a severe diabetogenic environment.

A close-up view of the data sets for Pima Indian #5 and #7 exhibits that they experienced shorter periods of the diabetic outbreak than other patients. The steep upsurge of the glucose curves is underlined by the well-controlled glucose level before the outbreak. In particular, Pima Indian #5 spent only two years in developing severe diabetes from euglycemia, and this patient had been able to maintain a euglycemic state for 50 years before the outbreak. A scrutiny of the parameter values for all the patients reveals that lower values of *h*_1_ and *n*_1_ for Pima Indian #5 and #7, which represents lessened *X*-induced hepatic gluconeogenesis, may account for the well-managed glucose level before the diabetic outbreak. This highlights the importance of reducing hepatic gluconeogenesis for the treatment of hyperglycemia.

## Discussion

Mathematical models of diabetes progression play a crucial role in the study of the durable effect of anti-diabetic agents at different disease stages. However, few mathematical models have been proposed to investigate the long-course diabetes evolution owing to the inherent difficulty in capturing the complexity of diabetes. Even fewer models, if any, have been constructed to incorporate the impact of hostile environment (diabetogenic factors) on beta-cell dysfunction and the progression of diabetes, which hinders the quantitative study of the disease course with new treatment strategies. To alleviate this challenge, we established mathematical models studying beta-cell dysfunction and the disease evolution induced by potential pathogenic factors.

Based on the beta-cell mass regulation model of Topp et al. [1], we constructed a base model for the undisturbed glucose-insulin regulatory system. We revised the insulin-dependent glucose uptake in the Topp model by setting the insulin sensitivity as a decreasing function of insulin level to represent hyperinsulinemia-induced insulin resistance. The effect of glucose on functional beta-cell mass in the model has been replaced by the signal transduced by insulin, in view of the observation that there is no apparent elevation of glucose level to trigger beta-cell compensation during the prediabetic stage. Building upon the base model, we incorporated the environmental-induced or epigenetic-related diabetogenic factor, an inducement of beta-cell dysfunction, into the glucose regulatory system, formulating a general phenomenological diabetes progression model. The impacts of the pathogenic factor *X* on the dynamics of glucose, insulin and functional beta-cell mass are symbolized by incorporating the influence functions *p*_1_(*X*), *p*_2_(*X*), and *p*_3_(*X*) to the corresponding equations, representing the *X*-induced hepatic glucose production rate, insulin releasing rate and functional beta-cell mass net growth rate, respectively.

Numerical investigations of the obesity-related diabetes model illustrated the impact of the obesity-related factor on the progression of diabetes. For an individual exposed to a severe obesogenic environment, the result shows the obesity-related factor can induce the occurrence of hyperinsulinemia after about 7 years’ progression, the commencement of which precedes the outset of diabetes for almost 10 years. In addition, the insulin level of the patient starts to decrease in 19 years and insulin deficiency occurs over time subsequent to the appearance of beta-cell failure. The longtime glucose and insulin dynamics depicted by the model are in good agreement with the clinical observations of diabetes progression. We further studied the influence of controlled or eradicated obesity-related factor on the disease evolution. With the assumption of exposing the same individual to a mild obesogenic environment, the simulation result manifests apparent alleviation of the disease progression rate and severity. This supports the finding that weight loss of obese patients helps alleviate diabetes.

Analysis of varied upper bound values of the factor reveals an individualized threshold of the diabetogenic risk, separating the euglycemic and diabetic states. When the value of the obesity-related pathogenic factor stays below the threshold, the individual can stay euglycemic with certain degree of obesity. In contrast, an increasing excess of the threshold leads to the deterioration of diabetes. Such individualized threshold values, which provide warning signs of obese levels for taking necessary interventions to prevent the commencement of diabetes, may be valuable for clinical decision making. Although it may seem obscure how to quantify the obesity-related factor clinically, a closely associated concept of personal fat threshold (PFT) has already been utilized to design diet plans for diabetic patients [7, 55]. The PFT is determined by the extent of intra-pancreatic and intra-hepatic fat accumulation, as well as the individual sensitivity to local biochemical effects of superfluous lipids [55]. This perspective is proposed upon one of the clinical observations that the reversal of recent onset T2D can be achieved with a reduction of the pancreas and liver fat, even for nonobese people with T2D, by following a weight loss dietary regimen [56]. Overall, the quantification of the obesity-related factor can be accomplished feasibly with personalized clinical indices.

Bariatric surgery which corresponds to the case of an eradicated obesity-related factor in our model, has the remarkable and durable ability to reverse diabetes. Sherman et al. simulated the metabolic consequences of bariatric surgery, with certain assumptions on the parameters of hepatic glucose production, beta-cell function, and insulin sensitivity in the model [5]. Their model was constructed with the hypothesis that the beta-cell mass would increase initially to compensate for insulin resistance, which differs from our proposition that hyperinsulinemia is the upstream of insulin resistance for obesity-related diabetes. Our obesity-related diabetes model also successfully depicted the effects of RYGB surgery on the prompt reversal of diabetes, with an assumption that the diabetogenic factor would be eliminated quickly after the surgery. This highlights the potential of utilizing our model for clinical decision-making.

The underlying mechanism causing the ethnic disparities in the risks of diabetes can also be demonstrated by our obesity-related diabetes model. A higher insulin resistance and up-regulated beta-cell function in certain racial groups are suggested to elevate the risk of T2D [54]. We investigate the altered *GIβ* dynamics for a patient when the insulin resistance level and the up-regulating impact of the obesity-related factor on beta-cell function are lowered. The simulation result demonstrated that under the same obesogenic environment, this patient experienced an ameliorated disease state, and the time for the patient to step into the diabetic stage was delayed for longer than 11 years. Further investigations exhibited that the threshold value of the factor for this patient developing diabetes increases by three folds, indicating that the lower insulin resistance and reduced up-regulated beta-cell response may increase the tolerance of an individual to the obesogenic environment. Moreover, when this patient is exposed to a mitigated obesogenic environment, euglycemia (Fig 7) can be achieved successfully with tolerable beta-cell up-regulation. This is in line with the clinical observation that not all individuals with basal hyperinsulinemia would develop diabetes.

We performed sensitivity tests on the key parameters in our obesity-related diabetes model and the results demonstrated the robustness of the model. The analysis of the biological significance for these parameters also sheds light on the possible efficient treatment strategies. The parameters *h*_1_, *q*_1_, *q*_2_, *n*_1_ and *n*_2_ have been shown to carry primary influence on the disease progression. The decreased value of *n*_1_ and *n*_2_, corresponding to the alleviated severity of the obesity-related factor from the hostile environment, can delay or even prevent the diabetes progression. This demonstrates the benefit of controlling the diabetogenic factors to the treatment of diabetes, as presented in the clinical data [56]. Likewise, the lower value of *q*_1_, representing a lower beta-cell susceptibility towards the pathogenic factor, contributes to slowing down or preventing the diabetes progression. In contrast, the increase of *q*_2_, representing improved tolerance of beta-cells to the factor, can retard the disease evolution. Therefore, the approaches that desensitize the beta-cells to diabetogenic factors can be potent for diabetic therapy. Furthermore, the decline of *h*_1_, which stands for the reduction of the pathogenic factor-induced hepatic gluconeogenesis, can slow down the diabetes progression and abate the final steady state of glucose level. Nevertheless, decreasing the value of *h*_1_ alone cannot prevent the development of hyperglycemia, which implies the monotherapy of suppressing gluconeogenesis is not sufficient to cure diabetes.

The effects of reduced values of *h*_1_, *q*_1_, *n*_1_ on the control and prevention of diabetes were exemplified by the model fitting to the Pima Indian data. The overall results from the simulation and data fitting indicate that the disease prevention can be achieved by ameliorating the diabetogenic environment or decreasing the susceptibility of beta-cells to the environment. The monotherapy of reducing hepatic gluconeogenesis or insulin resistance can retard the disease evolution, yet is inadequate to halt diabetes progression. Nevertheless, the Roux-en-Y surgery, which eradicates both the factor-induced hepatic gluconeogenesis and beta-cell dysfunction, has shown fast and lasting therapeutic effects on the reversal of diabetes.

By fitting the obesity-related diabetes model to the longitudinal T2D data of the Pima Indian tribe [30], we obtained individualized parameter values for the patients, as well as the predicted glucose trajectories over their lifespan. The best fits well delineate the trend of glucose variations presented in the data. Each outbreak period displayed in the data sets was sufficiently captured by our fits. In addition, the distinct characteristics among the data sets were able to be apprehended by the differences of estimated parameter values, which confirms the biological significance of the parameters. All of these highlight the feasibility of applying our model to studying the diabetes progression clinically. The anticipated glucose trajectories may also assist clinicians in determining the optimal treatment strategies at different diabetic stages. In particular, intensive therapy, such as bariatric surgery, could be an option for patients in the severe diabetic stage when moderate interventions, like medication treatment, are often insufficient. With the predicted progression trajectories, our model may help physicians decide the optimal time to apply such surgery for an ideal prognosis.

Although our model provides a good fit to the longitudinal data of Pima Indian patients, there remains unclarity whether hyperinsulinemia or insulin resistance comes first. Incorporating the impact of hyperinsulinemia on insulin resistance in the model is important to investigate the diabetes progression for patients who develop hyperinsulinemia prior to insulin resistance. In the first simulation (Fig 2), we assume the insulin sensitivity function *C*(*I*) depends only on the insulin level. The limitation of this assumption is that insulin sensitivity can become greater than its initial value when insulin decreases to below a certain level. Assuming the insulin sensitivity function also depends on the obesity-related factor *X* can overcome this limitation (Fig 3A). However, introducing a function of *X* to the insulin sensitivity function involves more parameters, bringing more challenges to data fitting. Of note, we did not observe a significant change in the glucose-insulin dynamics using the two functions *C*(*I*) and *C*(*I*, *X*) (Fig 3B–3D). Thus, we used the function *C*(*I*) with fewer parameters in model fitting to the Pima Indian patients. In view of the limited data, we also found it challenging to obtain precise estimates of all the parameters in our models. Some of the parameter values that guarantee the consistency of simulated glucose-insulin dynamics with clinical observations were adopted, as we aim to establish phenomenological models describing the diabetes progression, instead of providing precise estimates of all the parameters. The measurement of beta-cell mass itself is intractable, hampering optimal parameter calibrations for all established models in the literature incorporating the beta-cell mass. Hopefully, future development of measurement technology in this field can provide handy data for further model validation.

In summary, we established a novel mathematical model that introduces a general pathogenic factor into the glucose regulatory system. The obesity-related diabetes model, capable of characterizing the clinical feature of disease progression, provides a promising framework for disease intervention and individualized patient treatment. Although the impact of the diabetogenic factor on the diabetes progression was investigated in detail with the factor specified to be obesity-related, the generalized diabetes progression model may also provide a feasible framework to study how the glucose regulatory system is influenced by other pathogenic factors as well, such as the parasecretion of thyroid hormones and epinephrine.

## Supporting information

S1 Fig Sensitivity test on parameters *h*_1_, *α* in the obesity-related diabetes model and the influence of *h*_1_ on diabetes progression. (TIFF)

S2 Fig Sensitivity test on parameter *h*_2_ in the obesity-related diabetes model and the influence of significantly elevated *h*_2_ on the *GIβ* dynamics. (EPS)

S3 Fig Sensitivity test on parameters *q*_1_, *q*_2_ in the obesity-related diabetes model, and the influence of *q*_1_ on diabetes progression. (TIFF)

S4 Fig The influence of parameter *n*_1_ on the severity of factor *X* and diabetes progression. (EPS)
