# Supplementary figures and images for: Modeling the progression of Type 2 diabetes with underlying obesity
Source: PLoS Comput Biol. 2023 Feb 27;19(2):e1010914. doi: 10.1371/journal.pcbi.1010914 (PMC9997875; doi:10.1371/journal.pcbi.1010914)

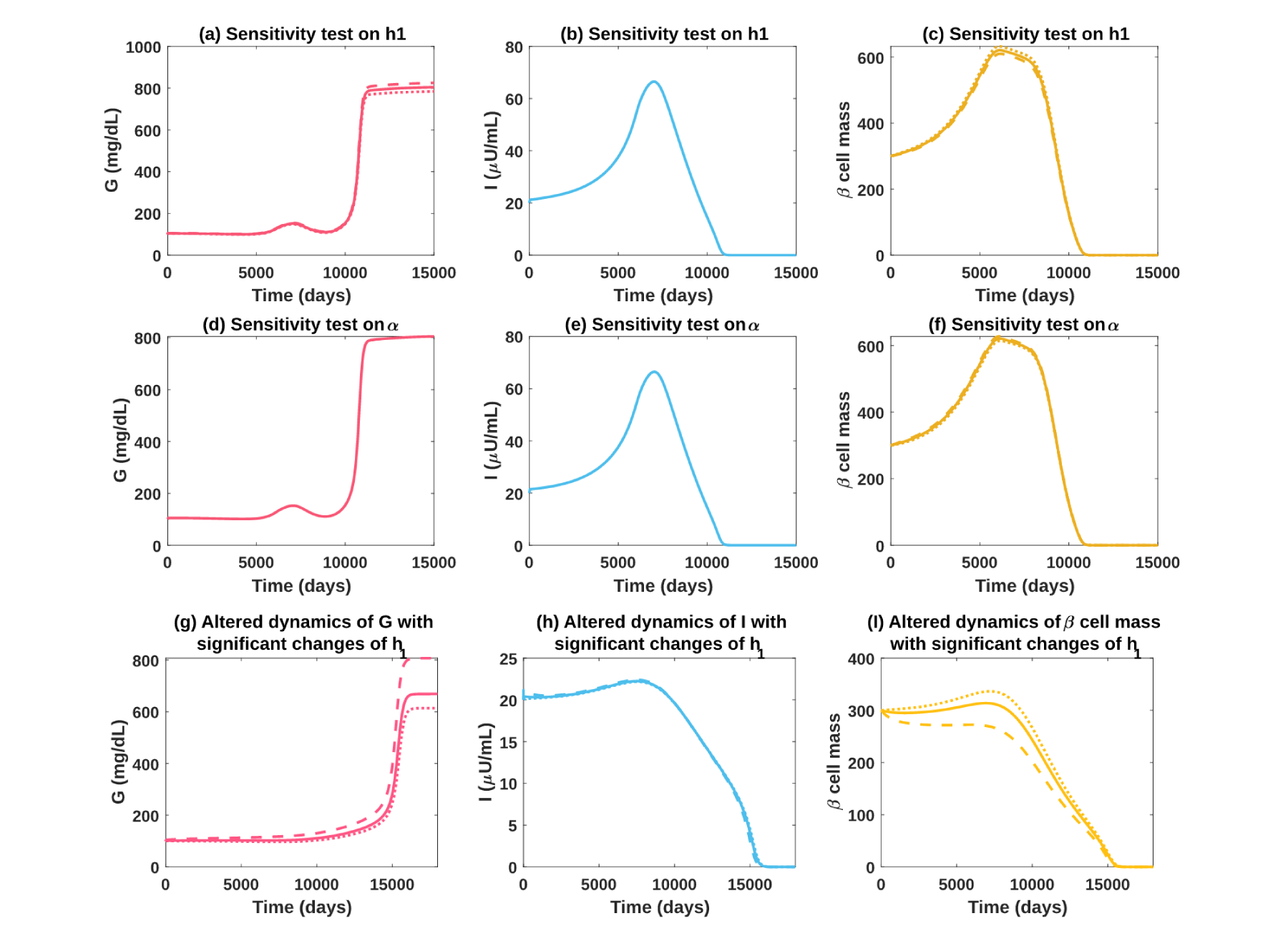

Supplement: S1 Fig — (TIFF) [file pcbi.1010914.s001.tiff]

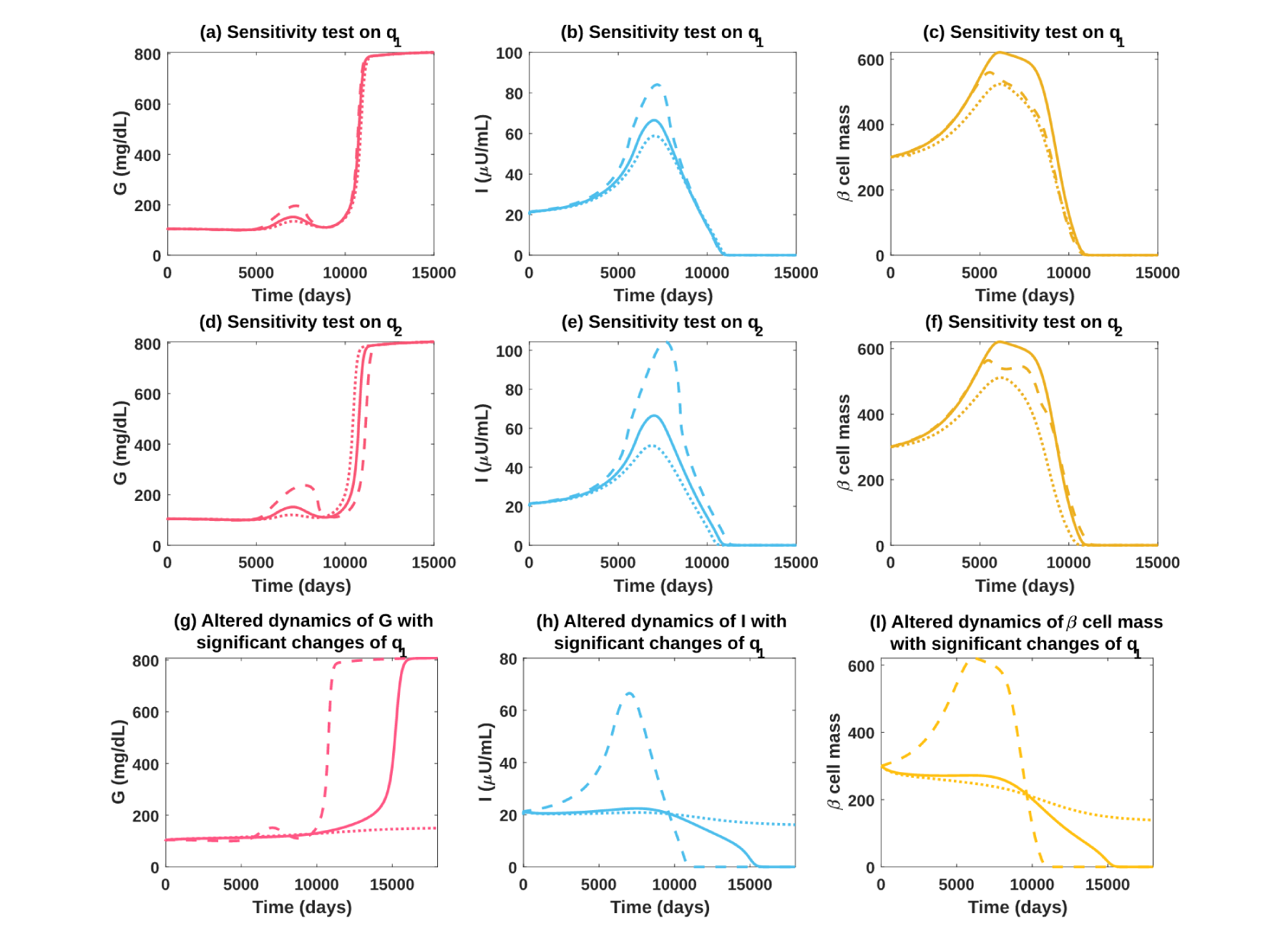

Supplement: S3 Fig — (TIFF) [file pcbi.1010914.s003.tiff]
